# Sepsis-associated brain dysfunction in critically ill patients

**DOI:** 10.1186/cc12929

**Published:** 2013-11-05

**Authors:** Cristiane Damiani Tomasi, Franciele Vuolo, Larissa de Souza Constantino, Dhébora Mozena Dall'Igna, Eduardo Mazon, Renato Mafioleti, João Quevedo, Cristiane Ritter1, Antonio Teixeira, Felipe Dal-Pizzol

**Affiliations:** 1 Laboratório de Fisiopatologia Experimental and Instituto Nacional de Ciência e Tecnologia Translacional em Medicina, Programa de Pós-Graduação em Ciências da Saúde, Unidade Acadêmica de Ciências da Saúde, Universidade do Extremo Sul Catarinense, 88806-000, Criciúma, SC, Brazil; 2 Departamento de Bioquímica e Imunologia, Instituto de Ciências Biológicas da UFMG, Belo Horizonte, MG, Brazil

## Background

Delirium is a common occurrence in critically ill patients and is associated with an increase in morbidity and mortality [1]. Some evidence suggests that septic patients with delirium may differ from a general critically ill population. In a subgroup analysis of the MENDS study, a benefit of dexmedetomidine sedation over lorazepam was only evident in septic patients [2]. The aim of our study was investigate the relationship between systemic inflammation and the development of delirium in septic and nonseptic critically ill patients.

## Materials and methods

We performed a cohort study in a 20-bed mixed ICU that included consecutive patients admitted for more than 24 hours. Delirium was diagnosed using the Confusion Assessment Method for the Intensive Care Unit (CAM-ICU). Coma was defined as a Richmond Agitation Sedation Scale (RASS) score of -4 or -5. Blood samples were collected within 12 hours of enrollment for determination of TNFα, soluble TNF receptor (STNFR)-1 and STNFR-2, IL-1β, IL-6, IL-10 and adiponectin.

## Results

Seventy-eight patients were included in the study: 26 nonseptic/nondelirium (control), 13 nonseptic/delirium (delirium), 21 septic/nondelirium (septic) and 18 septic/delirium (sepsis-associated delirium (SAD)). From all analyzed biomarkers only STNFR1, STNFR2 and adiponectin were independently associated with delirium occurrence, but none of these biomarkers had a significant interaction with sepsis. In contrast, there was significantly interaction between sepsis and IL-1β suggesting that this cytokine is differently modulated when comparing septic and nonseptic patients with delirium.

## Conclusions

The association between IL-1β and delirium is different in septic versus nonseptic patients, suggesting that mechanisms which drive SAD may differ from that of nonseptic ICU delirium.
